# Case of Amyloid Degeneration of the Liver and Kidneys, with Caries of Lumbar Vertebrae

**Published:** 1877-08

**Authors:** W. T. Belfield

**Affiliations:** Ass’t Physician, Cook Connty Hospital


### CASE OF AMYLOID DEGENERATION OF LIVER AND KIDNEYS, WITH CARIES OF LUMBER VERTEBRAE.

By Dr. W. T. BELFIELD, Ass't Physician, Cook County Hospital.

Mary L., aged 3 years, was admitted to the hospital, May 1, 1875. For some reason which can be only conjectured, neither her previous nor her subsequent history was recorded; hence our only information in regard to her, is gleaned from the traditions handed down to the present internes, and from the personal knowledge of the nurse. Unsatisfactory as such a history necessarily is, the rarity of the case is, perhaps, sufficient apology for its presentation.

The child had been always delicate; had never walked, the inability being due, apparently, not to malformation, but to a lack of strength. On admission she was quite emaciated; skin cool, very white and transparent; appetite excellent, usually had several clay-colored stools daily; from a small opening below Poupart's ligament on the right side there was a slight but constant purulent discharge; there was evidently considerable liquid in the peritoneal sac. She was so weak as to be unable even to feed herself; her mental faculties were quite acute, and she took the usual delight in the ownership of dolls and other toys.

During the first year, her history was simply a gradual aggravation of the ascites, and of the diarrhœa; her stools finally averaging from 12 to 20 daily, but retaining their peculiar clay color and semi-solid consistence. Some enlargement of the liver became evident. During all this time, notwithstanding the unavoidable lack of exercise, society and amusement, her general condition did not decline; her appetite became voracious; and except during periodical exacerbations of the diarrhœa, she complained of no pain. She was serene, contented, almost happy. The late Prof. Freer, upon taking charge of the ward in which she lay, pronounced the case one of amyloid degeneration of the liver.

About a year ago a small tumor appeared and burst in the gluteal region of the right side. From this opening there was a constant purulent discharge. About six months ago, the abdomen being then greatly distended, there became apparent œdema of the feet, gradually extending upward to the knees. From this time the little patient declined visibly in health, and finally, without the development of additional symptoms, died May 2, 1877.

A *post-mortem* examination revealed the presence of about five quarts of clear, yellowish liquid in the peritoneal sac; a fibrillated exudate on the superior surface of the liver, and among the folds of intestine. The liver was much enlarged, weighing 57 ounces; was very smooth, dense and elastic; the edges were rounded; the cut surface was of waxy appearance, dry and almost bloodless; the lobules were plainly mapped out by white boundary-lines; a thin section was quite translucent; the addition of iodine in solution produced a mahogany-red color.

The kidneys, also, were enlarged, smooth and dense, weighing 3 ounces each. Section showed the enlargement to be due chiefly to thickening of the cortical portion, which also presented an unusual pallor. Treatment with iodine caused the malpighian bodies and vasa recta to assume the same mahogany-red color.

There was also extensive caries of the lumbar vertebra, from which a sinus extended to the opening on the hip.

Microscopic examination was made under the supervision of Dr. Danforth, pathologist to the hospital. Sections were cut by hand, soaked for a few minutes in glycerine and acetic acid, and then stained with a solution of iodine in iodide of potassium and water. In the liver, the calibre of the interlobular vessels appeared somewhat diminished; the liver-cells were swollen, of rounded, regular outline, and devoid of nuclei; in many cases there seemed to have been obliteration of the intercellular substance and coalesence of cells; the entire lobule, excepting only the scanty connective tissue, presented the mahogany-red color. There was no evidence of fatty degeneration.

In the kidney, the vascular coils of many malpighian bodies, the corresponding vasa afferentia, and some of the vasa recta, exhibited the iodine staining. Some of the malpighian bodies and many vasa recta showed no change of color.

This case is in accord with the published statements of authorities. Thus Niemeyer says: "Lardaceous liver never occurs in persons otherwise healthy; it is more apt to occur in advanced cachexia, particularly in cases resulting from scrofulous or syphilitic affections, from tedious suppurations, and caries of bone."

Rindfleisch inverts the order of probable causes, stating that it is found "most frequently after long-continued suppuration in the osseous system, caries of the vertebræ, necrosis, etc.; furthermore, it is not a rare attendant upon constitutional syphilis." Green gives still more prominence to the predisposing influence of suppuration, stating that the disease most frequently occurs in conjunction with "profuse and long-continued suppuration, such as chronic diseases of bone, empyema," etc. "It also frequently occurs in the advanced stages of constitutional syphilis, but usually only in those cases in which there is chronic bone disease or chronic ulceration."

Judging from our scanty history, and from the microscopical appearances, it appears probable that the patient in question was of scrofulous diathesis; that the first lesion was caries of the vertebræ; that this was soon followed by amyloid infiltration of the liver, and finally, probably within eight months of the date of her death, by a similar degeneration of the kidneys.

Reported May 28, 1877.

